# Systematic Identification and Evolution Analysis of *Sox* Genes in *Coturnix japonica* Based on Comparative Genomics

**DOI:** 10.3390/genes10040314

**Published:** 2019-04-22

**Authors:** Lan Jiang, De Bi, Hengwu Ding, Xuan Wu, Ran Zhu, Juhua Zeng, Xiaojun Yang, Xianzhao Kan

**Affiliations:** 1 The Institute of Bioinformatics, College of Life Sciences, Anhui Normal University, Wuhu, 241000, China; jianglan@ahnu.edu.cn (L.J.); bide1125@vip.163.com (D.B.); wuxuan@ahnu.edu.cn (X.W.); zhuran0911@ahnu.edu.cn (R.Z.); j.h.zeng@ahnu.edu.cn (J.Z.); 2 State Key Laboratory of Genetic Resources and Evolution, Kunming Institute of Zoology, Chinese Academy of Sciences, Kunming, 650000, China; 3 The Provincial Key Laboratory of the Conservation and Exploitation Research of Biological Resources in Anhui, Wuhu, 241000, China; hengwuding@ahnu.edu.cn

**Keywords:** SOX, gene family, HMG-box, Japanese quail, genomics

## Abstract

*Coturnix japonica* (Japanese quail) has been extensively used as a model animal for biological studies. The *Sox* gene family, which was systematically characterized by a high-mobility group (HMG-box) in many animal species, encodes transcription factors that play central roles during multiple developmental processes. However, genome-wide investigations on the *Sox* gene family in birds are scarce. In the current study, we first performed a genome-wide study to explore the *Sox* gene family in galliform birds. Based on available genomic sequences retrieved from the NCBI database, we focused on the global identification of the *Sox* gene family in *C. japonica* and other species in Galliformes, and the evolutionary relationships of *Sox* genes. In our result, a total of 35 *Sox* genes in seven groups were identified in the *C. japonica* genome. Our results also revealed that dispersed gene duplications contributed the most to the expansion of the *Sox* gene family in Galliform birds. Evolutionary analyses indicated that *Sox* genes are an ancient gene family, and strong purifying selections played key roles in the evolution of *CjSox* genes of *C. japonica.* More interestingly, we observed that most *Sox* genes exhibited highly embryo-specific expression in both gonads. Our findings provided new insights into the molecular function and phylogeny of *Sox* gene family in birds.

## 1. Introduction

The *Sox* gene family is defined as a sex-determining region Y (SRY)-related high-mobility group (HMG) box of genes [1]. According to the similarity of HMG domain sequences and structural characteristics, the *Sox* subfamily is clustered into at least 11 groups (SoxA–K) [2]. The amino acid identity of the same *Sox* group is usually at least 70% [3]. Groups B–F are found in all vertebrate as core groups [4]. The SoxB gene is the one that duplicated the most recently into SoxB1 and SoxB2, and *Sox15* (SoxG) descended from *Sox19* (SoxB1) during vertebrate evolution [5]. Interestingly, these core groups probably underwent two duplication and divergence events, often resulting in three closely related *Sox* genes [3,6]. Non-core groups are lineage-specific, including SoxA in mammals [7], SoxI and SoxJ in *Caenorhabditis elegans* [1], and SoxK in medaka [8]. Notably, SoxH genes, although previously described as mammalian-specific, have been identified in invertebrates [4]. These non-core groups were considered to have originated from core *Sox* genes by tandem duplication events and gene losses, e.g., the *SRY* gene (group A) from *Sox3* (group B1), and *Sox30* (SoxH) from SoxD [4,5]. Furthermore, subfunctionalization and neofunctionalization are the main reasons for keeping duplicated genes under the selective pressure of evolution [3]. The presence of *Sox* genes in distantly related species indicates a strong selective pressure on their functional activity.

To date, about 10 *Sox* genes have been identified in invertebrates, and about 40 *Sox* genes have been found in vertebrates [9]. Nine *Sox* genes were detected in *Bombyx mori*, belonging to five groups of B–F [10]. In humans and mice, there are 20 members, belonging to nine groups (Sox A, B1, B2, C–H) [10]. *Sox* genes also exist in some other animals, including 20 presented in catfish [11], 13 in sea gooseberry [12], eight in fruit fly [13], and five in nematode [13]. With the identification and further annotation of the *Sox* gene family, researchers have found that *Sox* genes play important roles in diverse growth and development processes, such as chondrogenesis, neurogenesis, early embryonic development, and sex determination and differentiation [14]. For example, *Sox9* plays an essential role for the survival and proper proliferation of medaka germ cells [15]. *Sox7* and *Sox18* have been verified to contain redundant roles in arteriovenous specific cation and vascular development in zebrafish [16].

Over the last decade, along with the rapid development of sequencing technologies, the genomic and genetic resources for birds are expanding massively [17,18,19,20,21,22,23]. Now, 12 genome sequences from galliform birds are available from the NCBI database, and eight of which were used in this study, including bamboo partridge [24], chicken [25,26], turkey [27], greater prairie chicken (unpublished), helmeted guineafowl (unpublished), Japanese quail [28], northern bobwhite [29], and scaled quail [30]. Genomic information would be instrumental in helping to understand the *Sox* gene family in birds. In comparison to mammals, birds have a different sex-determining system (ZZ for males and ZW for females) [31]. This provides a good opportunity to analyze the *Sox* gene family from a new perspective. Some members of the *Sox* gene family have been found in birds from the earlier reports. For example, *Sox9* cDNAs were isolated from duck and quail (five days and seven days in the gonads) [32]. In several neural crest-derived cell types, the expression of *Sox9*, *Sox10,* and *Sox5* could activate that of *Col2a1* [33]. *Sox9* in chicken was observed in seven-day testes with high level expression, while *Sox3*, *Sox4*, *Sox8*, and *Sox11* had similar expression levels in both gonads [12]. However, genome-wide investigations of the whole *Sox* gene family in birds are still largely unknown.

*Coturnix japonica* (Japanese quail), which belongs to the Galliformes order [34], is a tiny species of Old World quail found in East Asia [35]. It is widely used for meat and egg productions [36]. Moreover, similar to chicken, goose, duck, and pigeon, quail is also easy to rear and has been used extensively as a model animal for biological studies [37,38,39,40]. Due to its economic value, the genome and transcriptome of *C. japonica* were recently sequenced, laying a solid foundation for the comprehensive analysis of the *Sox* gene family in *C. japonica*. The aim of this study was to identify the members of the *Sox* family in *C. japonica* and compare them with other Galliformes species to explore the evolutionary relationship of *Sox* genes among different species.

In the current study, we performed a genome-wide study to explore the *Sox* gene family in galliform birds. We carried out a phylogenetic analysis of the *Sox* gene family that included performing structural characterization, studying protein–protein interaction (PPI) and conserved microsynteny, and documenting their large-scale duplication and the influence of strong purifying selection. Furthermore, we also assessed the expression patterns of *CjSox* genes in gonads. Our findings provided new insights into the molecular function and phylogeny of the *Sox* gene family in birds.

## 2. Materials and Methods

### 2.1. Ethics Statement

The animal experiments in the current study were authorized by the Ethics Committee of Anhui Normal University (Anhui, China) (No. 2018016). All the birds used were sacrificed following a procedure minimizing their suffering as much as possible.

### 2.2. Incubation Conditions

*C. japonica* was reared at the animal room environment of Anhui Normal University (Wuhu, China). All the fertilized eggs used in this study were incubated at 38 °C, with 55–65% relative humidity in the fully automatic egg incubator (WQ03 mini egg, by Tongda device manufactory, Dezhou, China) for 15 days. All the quails in the whole experiment were housed in laying cages (90-cm wide, 40-cm deep, 33-cm high, 30 quails per cage), and fed with basal diets. The gonads of incubation period (day 14) and post-hatch period (day 14) quails were chosen for Q-PCR analysis. Here, the incubation period refers the time from the start of uninterrupted incubation to the emergence of the young, and the post-hatch period means the time after quails hatch out of the shell [41].

### 2.3. Retrieval of Genome Sequences

The genomic sequences of *C. japonica* and the seven other available Galliformes species were employed for comparative analyses in this study. All the data was downloaded from NCBI (https://www.ncbi.nlm.nih.gov/genome). Three strategies were applied to identify *Sox* genes from the above genome sequences. First, the released SOX protein sequences of *G. gallus* were used to query putative SOX homologous proteins using BLASTp program [42] with e-value cutoff 1e-5. Subsequently, the conserved HMG-box domain (InterPro ID: IPR009071) of 79 amino acids was searched against the local database by the local BLASTp program, with an e-value threshold of 1e-4. Irrelevant sequences were deleted manually. Finally, the Pfam and SMART databases were utilized to check the candidate sequences that contained HMG-box domains (InterPro ID: IPR009071).

The data of chromosome locations, CDS (coding sequence) lengths, and number of amino acids were derived from NCBI. The theoretical molecular weight (kDa) and pI (isoelectric points) of each SOX protein were calculated by the pI/MW tool (http://www.expasy.org/tools/). GRAVY (grand average of hydropathy) values were evaluated using the PROTPARAM tool (http://web.expasy.org/protparam/). MCScanX v1.0 was employed to detect the duplicated type and collinear blocks according to a previous report [43].

The DNA and cDNA sequences, corresponding to each predicted gene from the genomes, were downloaded from NCBI. MEME (Multiple Em for Motif Elicitation) version 5.0.2 [44] was used for predicting the conserved motif structures encoded by quail and chicken *Sox* genes. The GFF (general feature format) files were obtained from the NCBI database, and the R software was used to parse GFF3 files and map *Sox* genes onto chromosomes [45].

### 2.4. Phylogenetic Analysis

To study the evolutionary origin of the *Sox* genes in galliform birds, two data sets were executed as follows. (1) The tree of the eight Galliform species was downloaded by obtaining data from NCBI Common Tree. (2) All the sequences of predicted HMG-box domains from eight galliform species and lizard were aligned by ClustalW in MEGA X with default parameters [46], using the maximum likelihood (ML) method with 1000 bootstrap replicates by RaxML v8.2 (ML) [47], set MmTCF1 as an outgroup [48]. The constructed tree files were visualized using Figtree v1.4.4 (http://tree.bio.ed.ac.uk/software/figtree/) and iTOL v4.2.3 [49].

### 2.5. Microsynteny, Selection, and Functional Analysis

The microsynteny analysis was used to classify and identify the expansion pattern of the *Sox* gene family. Three files, including the gene identifier file, gene list file, and CDS file, were generated to carry out the microsynteny analysis. Firstly, all the *CjSox* genes were located in the *C. japonica* genome as an anchor point. Then, we analyzed the protein-coding sequences within 20 genes downstream and upstream of each anchor point using the BLASTp program [42]. In the present study, we defined a syntenic block as a region where at least two conserved gene pairs were located within 20 genes downstream and upstream between genomes, and this syntenic block was considered to have originated from a large-scale duplication event [50]. The ratio of non-synonymous (Ka) to synonymous (Ks) substitution rates (Ka/Ks) was calculated for coding sequences with DnaSP v6 [51]. Then, FEL (Fixed Effects Likelihood) was used to select individual codons by DataMonkey [52]. PPI (protein–protein interaction) data was obtained from the online database STRING [53]. GO enrichment analysis was performed by clusterprofiler [54].

### 2.6. RNA Extraction, Q-PCR, and RNA-Seq Expression Analysis

Total RNAs were isolated from the ovaries and testes using TRIzol reagent (Sangon Biotech Co. Ltd., Shanghai, China). The first-strand cDNA was synthesized from the RNA using a PrimeScript RT reagent Kit with gDNA eraser (TaKaRa, Otsu, Japan) according to the manufacturer’s instructions. Gene-specific primers were designed using Primer-BLAST (https://www.ncbi.nlm.nih.gov/tools/primer-blast/) (the details are shown in Table 1). For the Q-PCR reaction, 1 µL of cDNA was mixed with 10 μL of 2× SuperReal PreMix Plus, 1.6 µL of gene-specific primers (1.0 µM), and 7.4 µL of ddH_2_O in a PCR reaction mix (SuperReal PreMix Plus (SYBR Green), Qiagen, Tecnolab). Each treatment was carried out in triplicate with a reaction volume of 20 µL, and independently repeated three times. We conducted Q-PCR on the Bio-Rad CFX96 (Bio-Rad Laboratories, Inc., Hercules, CA, USA) with two-step methods, under the following conditions: 95 °C for 15 min, 40 cycles of 95 °C for 10 s, 60 °C for 32 s. We calculated the gene expression by the 2^−ΔΔCT^ method [55]. Three biological replicates were conducted for each sample. The RNA-seq raw data of two developmental stages (incubation and post-hatch periods at 14 days, respectively) were used in quails (unpublished data from our group), and the concrete methods were shown in the previous report [45,56].

## 3. Results and Discussion

### 3.1. Genome-Wide Analysis of Galliformes Sox Genes

**Figure 1:**
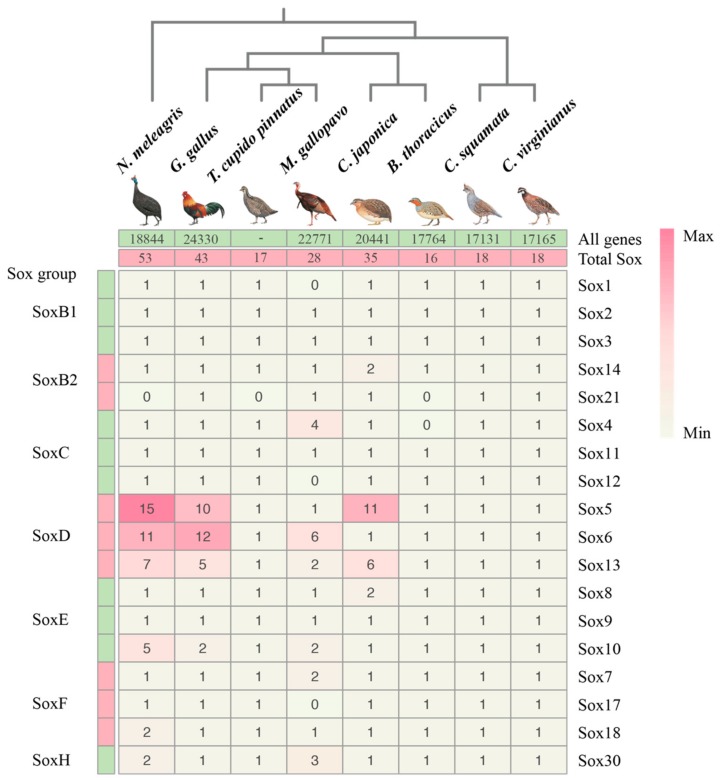
The phylogenetical analyses, categories, and abundance of *Sox* gene members in galliform birds. Note: The total gene number of *T. cupido pinnatus* genome is not available in NCBI.

In recent years, studies on the *Sox* genes family have been reported in many different animals [57]. However, the genome-wide identification and analyses of *Sox* genes from avian genomes has not been characterized in detail to our knowledge. In our study, a total of 35 *Sox* genes (Figure 1) were identified in the *C. japonica* genome, which had 20,441 genes in this genome. Among eight galliform genomes, three species (*Gallus gallus*, *C. japonica,* and *Numida meleagris*) had over 10 variants in *Sox5*, and there were over five variants in *Sox13*. These two genes (*Sox5* and *Sox13*) belong to the SoxD group. More noticeably, four species (*G. gallus*, *C. japonica*, *Colinus virginianus*, and *Callipepla squamata*) had 18 different *Sox* gene members, while the remaining species had less (Figure 1 and Table S1). For instance, *Sox1*, *Sox12*, and *Sox17* were missing for *Meleagris gallopavo*, *Sox4* and *Sox21* were missing for *Bambusicola thoracicus*, and *Sox21* were missing for *N. meleagris* and *Tympanuchus cupido pinnatus*. The total number of *Sox* genes of *C. japonica* (35) was less than those of *G. gallus* (43) and *N. meleagris* (53), and more than the other species (Figure 1). Based on their conserved domain structures, the identified *Sox* gene members of *C. japonica* were categorized into seven groups as follows: *Sox1*, *Sox2*, and *Sox3* in the SoxB1 subgroup; *Sox14* and *Sox21* in the SoxB2 subgroup; *Sox4*, *Sox11*, and *Sox12* in the SoxC group; *Sox5*, *Sox6*, and *Sox13* in the SoxD group; *Sox8*, *Sox9,* and *Sox10* in the SoxE group; *Sox7*, *Sox17,* and *Sox18* in the SoxF group; and *Sox30* in the SoxH group. Notably, four *Sox* groups (SoxA, SoxG, SoxI, and SoxJ) that are present in mammals [58] were missing in *C. japonica* and other galliform birds. The category and abundance of *Sox* gene members in different galliform birds are compared in Figure 1.

Gene duplications have contributed exclusively to the expansion of gene families [59]. We examined five types of gene duplications: singleton, dispersed, proximal, tandem, and WGD (whole-genome duplication) or segmental duplication by the MCScanX program (Table 2). Most *Sox* genes belonged to the dispersed duplication. Comparatively, the percentage of the dispersed duplication was 100% in *C. squamata* and *B. thoracicus*, 92.9% in *C. virginianus*, 72.2% in *C. japonica*, 69.2% in *M. gallopavo*, 66.7% in *G. gallus*, and 60% in *N. meleagris*. Our results revealed that dispersed gene duplications contributed the most to the expansion of the *Sox* gene family in Galliform birds.

Noticeably, we found that genomic data from assemblies at the chromosome level (*G. gallus*, *C. japonica*, *M. gallopavo*, and *N. meleagris*) show a significantly higher number of *Sox* genes (43, 35, 28, 53 versus 16, 18, 18, and 17 from those at the scaffold level). Similarly, tandem and WGD/segmental duplications have also observed exclusively on the genomes assembled at the chromosome level. Bias between genomic data from different assembled level might be present, and might have affected the results. To avoid the bias, more high-quality bird genomic data (assemblies at the chromosome level) will be needed in the future studies on the avian *Sox* family.

The physicochemical properties of *Sox* genes in Japanese quails were calculated with ExPASy. The length, putative molecular weights, and theoretical isoelectric points (pI) of SOX proteins in Japanese quail are summarized in Table 3. The length of SOX proteins ranged from 240 to 816 amino acids in quail, with putative molecular weights ranging from 26.7 to 90.6 kDa, and theoretical isoelectric points (pI) ranging from 4.92 to 9.96. *Sox* genes in the SoxD group contained a higher number of amino acid residues than other groups. All the members of the SoxB1 subgroup had a relatively low molecular mass. Ten SOX proteins had relatively low pIs (<7), including different members in SoxD, SoxE, SoxF, and SoxH, respectively. The other SOX proteins, particularly those in the SoxB group, had a pI >7. Compared with other galliform birds (taking *B. thoracicus* and *G. gallus* as examples) (Table S2) and channel catfish [11], the length in quail was higher than channel catfish (range from 242 to 805) and *B. thoracicus* (range from 240 to 797), and slightly lower than chicken (range from 240 to 817). By examining the properties of *Sox* genes for chicken (Table S2), we found that the length/pI/GRAVY/molecular weight of *Sox* genes was likely less varied in galliform birds. The grand average of hydropathy (GRAVY) score was calculated as the sum of the hydropathy values for all of the amino acids, divided by the protein length [60]. Unfortunately, all of the *C. japonica Sox* genes were hydrophilic, with GRAVY values <0. Our results indicated that most proteins in the same subfamily had similar parameters of physicochemical properties. This finding is congruent with the study carried out by Zhang et al. [11].

**Figure 2:**
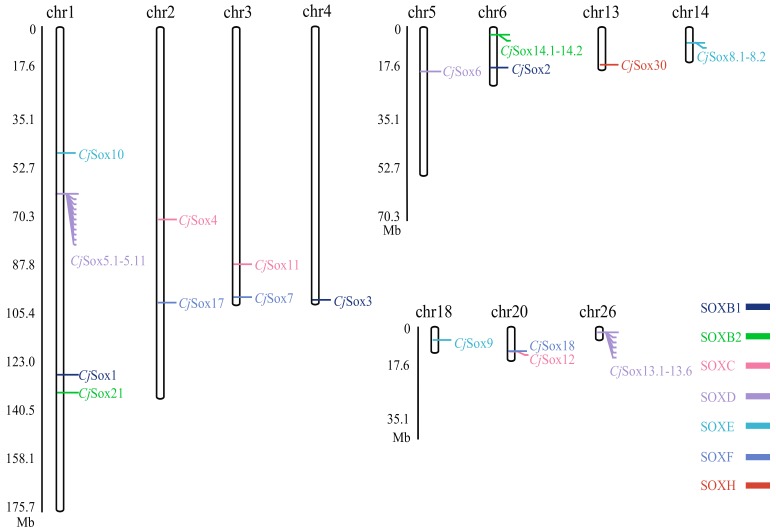
Distribution of the *Sox* gene family on quail chromosomes.

Furthermore, we determined the genomic positions of *Sox* genes in Japanese quail. These results indicated that the members of the *CjSox* gene family were randomly distributed across 11 of the 39 quail chromosomes (Figure 2). For example, there is only one *Sox* member located in chromosomes (chrs) 4, 5, 13, 14, 18, and 26, two *Sox* members located in chrs 2, 3, 6, and 20, and four *Sox* members (*CjSox1, 5, 10,* and *21*) located in chr1 (Figure 2). In contrast, no *Sox* members are present in the remaining chromosomes. This might be considered the result of interchromosome segmental duplication [11].

### 3.2. Phylogenetic and Classification of the Sox Gene Family

In order to gain insight into the relationships among species in Galliformes, we reconstructed a phylogenetic tree of the eight Galliform species. As shown in Figure 1, the common tree can be subdivided into three clades: *C. virginianus* and *C. squamata* belong to Odontophoridae; *G. gallus*, *C. japonica*, *M. gallopavo*, *B thoracicus,* and *T. cupido pinnatus* belong to Phasianidae; and *N. meleagris* belongs to Numididae. Interestingly, there was no positive correlation between the *Sox* genes from Galliformes genomes and the total number of genes contained in the corresponding genome. For instance, we found that there was no significant difference in the genome size of *C. japonica* (20,441) and *N. meleagris* (18,844); however, the *Sox* gene family numbers obviously changed. In contrast, the *Sox* genes in *C. virginianus* (17,165) and *C. squamata* (17,131) contain a positive correlation with the total number of genes contained in the corresponding genome.

**Figure 3:**
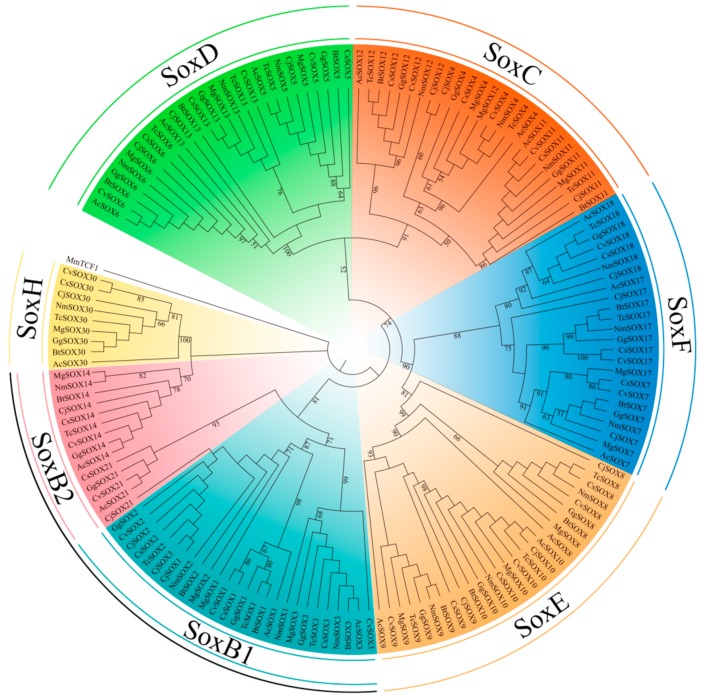
Phylogenetic tree of the Galliformes *Sox* family proteins. The tree was constructed using RaxML with the maximum likelihood (ML) method.

Then, we systematically identified the *Sox* genes by searching the local genome database of *C. japonica* and seven other representative species. After filtering, a total of 136 *Sox* genes were identified among Galliformes species (Figure 3). We further used the HMG-boxes from SOX amino acid sequences to construct an ML tree among the *C. japonica* and other species, including *Anolis carolinensis*, *C. virginianus*, *C. squamata*, *M. gallopavo*, *B thoracicus*, *G. gallus*, *T. cupido pinnatus,* and *N. meleagris*. According to the phylogenetic topology, we observed a relatively robust signal for a partition into five groups: SoxC (*Sox4*, *Sox11,* and *Sox12*, bootstrap value 91%), SoxD (*Sox5*, *Sox6,* and *Sox13*, bootstrap value 100%), SoxE (*Sox8*, *Sox9,* and *Sox10*, bootstrap value 81%), SoxF (*Sox7*, *Sox17,* and *Sox18*, bootstrap value 88%), and SoxH (*Sox30*, bootstrap value 100%). Remarkably, we also detected the closer evolutionary relationships between group D and group C, and group E and group F, indicating that they might have a common evolutionary origin during evolution.

Furthermore, as we know, SoxB genes have been identified from many bilaterian species [61]. It seems likely that SoxB genes are involved in neuroectoderm formation [61,62,63]. Interestingly, further studies in vertebrates revealed that SoxB1 and SoxB2 have opposite activities [9,61,64,65]. For instance, SoxB1 can be recognized by transcriptional activators, whereas SoxB2 act as repressors in the chicken [66]. Unfortunately, the reconstructed ML tree in this analysis also failed to recover two monophyletic SoxB groups (SoxB1 and SoxB2) (Figure 3), as did many previous reports [63,67,68,69].

### 3.3. Conserved Motif Analysis of Sox Genes

**Figure 4:**
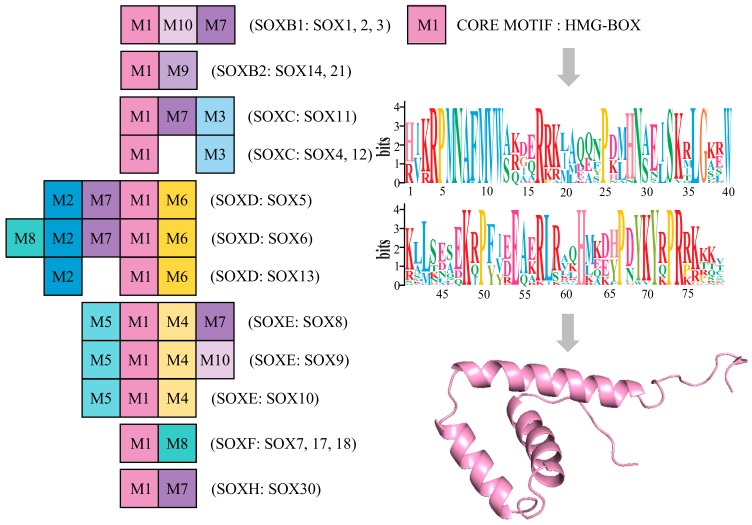
Motifs of the quail *Sox* family genes. Boxes with different numbers represent different motifs. M1 represents the core motif HMG-box (79 amino acids residues and the 3D structure are presented on the right).

Molecular studies have revealed that all the members of the *Sox* gene family encode transcription factors containing a highly conserved 79-amino acid motif (HMG-box) [48,70,71]. To further explore the origin and evolutionary pattern of *Sox* genes in *C. japonica*, conserved motif analysis was performed using the MEME program. The result of the current analysis indicated that 10 conserved motifs are presented in *C. japonica* (Figure 4). Noteworthily, we detected that conserved motif 1 (M1) exists in all the members of *Sox* gene family, while the other nine motifs are present in different *Sox* groups. As shown in Figure 4, three motifs (M1, M10, and M7) existed in the SoxB1 group, two (M1 and M9) existed in the SoxB2 group, and three (M1, M7, and M3) existed in the Sox11 group. SoxC contained M1 and M3; SoxD contained M1, M2, and M6; SoxE included M1, M4, and M5; SoxF contained M1 and M8; and SoxH contained M1 and M7. M1 is the core motif HMG-box, with 79 amino acids residues. The HMG-box is a type of DNA-binding domain that is found in a large number of eukaryotic proteins. The three-dimensional (3D) structure of M1 of *C. japonica* has a characteristic L-shaped fold, which is formed by three α-helices (Figure 4). We found the specific preservation and expansion of motifs in different *Sox* genes in *C. japonica*.

### 3.4. Gene Duplication of Sox Genes

**Figure 5:**
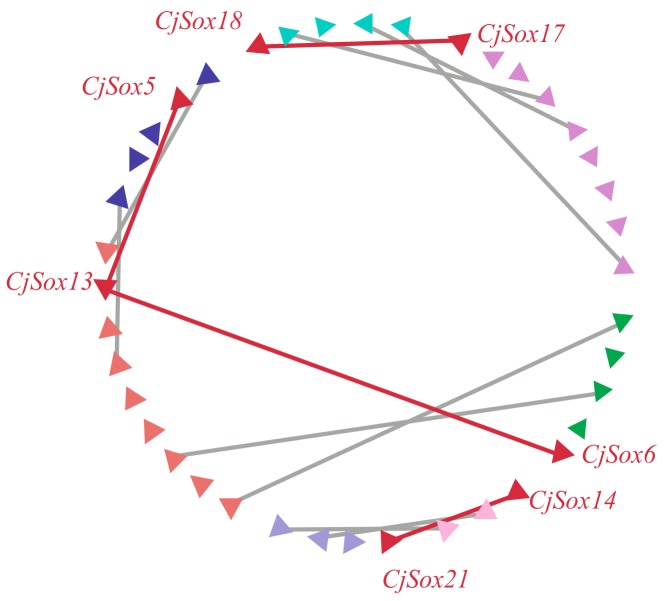
Microsynteny related to *Sox* genes in *C. japonica*. Triangles represented *Sox* genes and the flanking genes in the *Sox* gene family. The gene’s orientation is also represented by the triangle. The homologous genes on two fragments are connected by lines.

Normally, gene duplication is considered a contributor and an important source of biological evolution [50,72]. Two regions were generally considered to have evolved from duplication events when two or more protein-coding gene pairs flanking the anchor point had the best non-self hit (e-value < 1 × 10^−5^) [73,74]. To further understand how the *Sox* gene family evolved, we investigated gene duplication events of the *Sox* genes in *C. japonica.* The results of the analysis showed that 11 *Sox* genes were not present in any microsynteny, such as *CjSox1*, *CjSox2*, and *CjSox3*. This implied that these genes might be derived from duplication events. Additionally, we found significant microsynteny in the quail *Sox* gene regions. As shown in Figure 5, three conserved genes flanking in the *CjSox17*/*CjSox18* pair, and two conserved genes flanking in the *CjSox5*/*CjSox13*, *CjSox6*/C*jSox13*, and *CjSox14*/*CjSox21* pairs were identified, respectively. Our findings clearly indicate that four gene pairs were involved in the large-scale duplication during evolution of the *Sox* family in *C. japonica* (Figure 5), which is consistent with the published article by Voldoire et al. (2017) in teleost fish [75].

Ka/Ks < 1 suggests a functional constraint; a Ka/Ks ratio greater than one indicates a positive selection; a Ka/Ks ratio of one is neutral [76]. In the present study, we found that the Ka/Ks ratios of all the *CjSox* gene pairs were less than 0.6 (Table S3), suggesting that these gene pairs have evolved under strong purifying selection.

**Figure 6:**
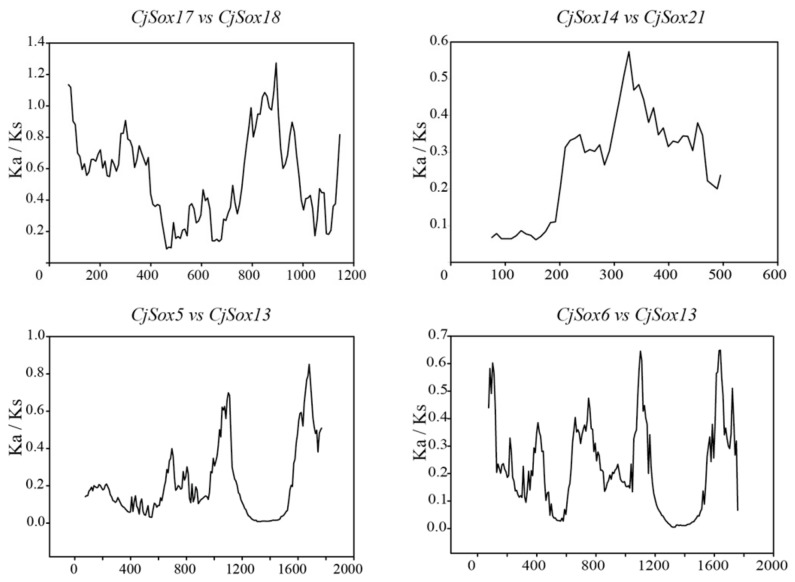
Sliding-window plots of representative duplicated *Sox* gene family members in *C. japonica*. The step size was 9 bp, and the window size was 150 bp.

It is worth noting that several codon sites could be masked by overall strong purifying selection during positive selection [77,78,79]. As a result, proteins with Ka/Ks values less than one may still contain sites under positive selection. Therefore, to better understand potential selection pressures in *C. japonica*, we carried out a sliding-window analysis of Ka/Ks between each pair of *CjSox* genes (Figure 6). As expected, except for several peaks (Ka/Ks > 1), the majority of Ka/Ks ratios across coding regions were far below one (valleys). The result demonstrated that strong purifying selections played a key role in the evolution of *CjSox* genes of *C. japonica.* Since the sliding-window analysis showed sites with Ka/Ks > 1, we then performed a thorough analysis to detect the signatures of positive selection in specific codons using the FEL analysis by the Datamonkey website [52]. The *Sox* genes of quail might have undergone positive selection during evolution for a few sites. We detected multiple positive sites relevant to the different *Sox* groups in quail for functional differentiation; the formation of multiple subgroups, where positive selection occurred are summarized in Table S4.

### 3.5. PPI Network Analyses of SOX Proteins and GO Enrichment Analysis

**Figure 7:**
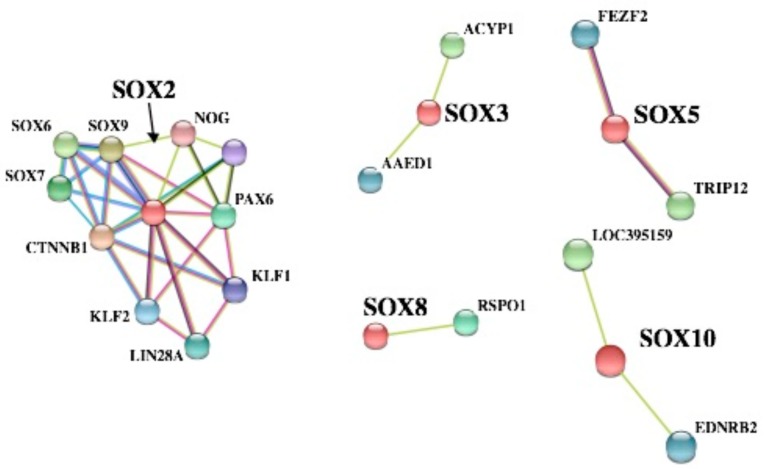
Predicted protein–protein interaction (PPI) network analyses of SOX proteins.

SOX proteins are involved in the differentiation of chicken germline stem cells, as they are in mammals [80]. We attempted to further analyze the PPI network of SOX proteins in *C. japonica* using the STRING database (version 10.5) (https://string-db.org/), and understand some potential functions by Gene Ontology (GO) enrichment analysis. The results exhibited five PPI network clusters by different colors (Figure 7). CTNNB1 (β-catenin) was reported as a crucial part in the epithelial cells of the chicken oviduct [81]. Our results clearly indicated that CTNNB1 is part of a complex network of interactions with SOX2, SOX6, SOX7, and SOX9. This finding indicates that these putative protein–protein associations might participate in embryogenesis and tumorigenesis in *C. japonica*. Additionally, ACYP1 was found to be involved in gastrointestinal stromal tumor cells [82], and AAED1 was a potential target for gastric cancer [83]. SOX3 might also be related to tumorigenesis in either a negative or positive manner [84]. In *C. japonica*, the network analyses also predicted that SOX3 was related to AAED1 and ACYP1; these interactions seem to be essential for treating gastric cancer. Moreover, we also found three other PPI networks of SOX proteins in *C. japonica* (Figure 7). For instance, SOX5 was integrated with FEZF2 and TRIP12, which might be essential for embryogenesis [85,86].

In order to further analyze the functions of the *Sox* gene family, we performed GO enrichment analysis (Table S5). The GO terms in this study included three categories: cellular component (CC, 3%), molecular function (MF, 20%), and biological process (BP, 77%). GO enrichment confirmed that part of these 18 *CjSox* genes are enriched in the nuclear transcription factor complex (GO:0044798), transcription factor complex (GO: 0005667), and nuclear part (GO:0044428) of the CC category. DNA-binding transcription factor activity (GO:0003700) and transcription regulator activity (GO: 0140110) were the most abundant functions in the MF category. The most enriched function was the regulation of transcription by RNA polymerase II (GO: 0006357). *CjSox9* exists in all 105 GO enrichment terms, suggesting that *CjSox9* plays important roles in the testis development, which is consistent with the findings of a previous study [32,87].

### 3.6. Gene Expression Analysis of C. Japonica Sox Genes in Gonads

**Figure 8:**
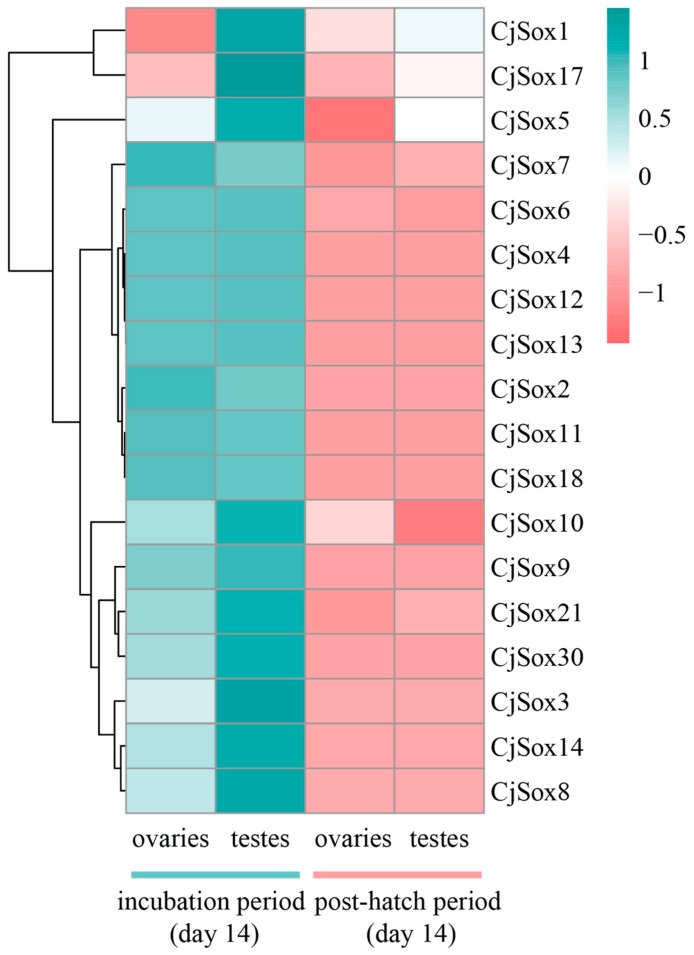
Heatmap of expression profiles for the *Sox* mRNAs in the quails. The color scale represents relative expression levels. The red or green color represented the higher or lower relative abundance of each transcript in each sample. Each column represents one sample, and each row indicates one *Sox* transcript.

**Figure 9:**
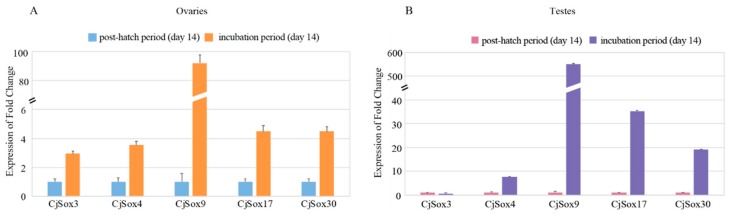
qPCR expression profile of the five quail *Sox* genes between the incubation period and the post-hatch period in the quail ovaries (**A**) and testes (**B**), respectively.

As reported in previous studies, multiple *Sox* genes were strongly expressed in the gonads and the central nervous system of mice [88,89,90,91], and widely expressed in different stages of embryogenesis [92]. However, so far, *Sox* genes expression in the gonads development of Japanese quail remains understudied. Thus, we further extracted the expression profiles (unpublished data from our group) for 18 *CjSox* genes between two developmental stages (incubation and post-hatch periods at 14 days, respectively) from quails with three biological replicates (Figure 8). Genome-wide gene expression analysis can potentially reveal period-specific expression manners for the *Sox* gene family in quail. SRY is associated with the development of testis, and the inhibition of SRY expression causes defects in testis development [93]. SRY is also directly targeted by *Sox9* [94], and previous studies have suggested that *Sox9* plays a key role in male gonad development in many vertebrates [95]. *Sox9* is down-regulated in the differentiating gonad just before ovary formation in chicken [96]. Notably, our result showed that one gene (*CjSox9*) was significantly more highly expressed in the incubation period (day 14) than in the post-hatch period (day 14) from both gonads (Figure 8, Figure 9 and Figure S1), implying that *Sox9* played a conserved role in the gonadal development of quails. Similarly, during the incubation period (day 14), three genes (*Sox5*, *Sox8*, and *Sox9*) were significantly more highly expressed in testes than in ovaries (*q*-value < 0.05). Furthermore, this investigation of the post-hatch periods in both gonads revealed that seven genes (*CjSox1, CjSox5, CjSox6, CjSox7, CjSox10*, *CjSox17,* and *CjSox21*) were weakly expressed, whereas the remaining 11 genes were negligibly expressed (Figure S1). We observed that most *Sox* genes exhibited highly embryo-specific expression in both gonads (Figure 8).

To further confirm the expression profiles, we carried out the qPCR experiment for five randomly selected *Sox* genes, including *Sox3, Sox4, Sox9, Sox17,* and *Sox30* (Figure 9). Our data suggested that these five *CjSox* genes presented similar expression patterns as RNA-Seq data. For example, four *CjSox* genes existed in both gonads, with similarly low expression levels for *Sox4, Sox9, Sox17,* and *Sox30* in the post-hatch period (day 14) of ovaries and testes as in the incubation period (day 14).

The *Sox* gene family was first considered to include the testes-determining genes, which were connected with gonadal development and the differentiation of gender [97,98]. Our findings in the current study suggested that most *Sox* genes are involved in gonadal development in *C. japonica*.

## 4. Conclusions

The *Sox* gene family plays an important role in developmental processes. Global identification and expression characterization of the *Sox* gene family were performed in *C. japonica* and compared with other species of Galliformes in this study. Here, we detected 35 *Sox* genes representing seven *Sox* gene subfamilies (SoxB1–B2, SoxC–F, SoxH) in quails. Based on homolog annotations and phylogenetic analyses, we named these *Sox* genes. The systematical analyses, including gene structure, conserved the HMG-box phylogenetic relationship, and microsynteny analysis suggested that these *Sox* genes were suitable for studying the evolution of the *Sox* gene family. The transcript analysis of *Sox genes* between the incubation period and the post-hatch period in the quail gonads showed that each *CjSox* gene had a unique expression pattern during sexual development. Expression profiling, PPI protein analyses, and GO analysis of these *CjSox* genes could provide the function characteristics of this gene family in Japanese quail growth and development.

## Figures and Tables

**Table 1:** Primers used in the qPCR analysis.

Gene Name	Primer Sequence (5′–3′)	Primer Length (bp)
Sox4-F	CATCAAGCGACCGATGAACG	20
Sox4-R	CTTCTCGCTGTCCTTCAGCA	20
Sox3-F	CCGAGATCAAGACTCCGCAA	20
Sox3-R	GTTCTCCTGGGCCATCTTCC	20
Sox9-F	GGAGAACACCCGACCTCAAG	20
Sox9-R	CGTGGGGTTTGTTCTTGCTG	20
Sox17-F	TAAATCGTGGAAGGCGCTGT	20
Sox17-R	GCCGCTTCACCTGCTTCTTC	20
Sox30-F	CTAGGATTCACCGACCTGCC	20
Sox30-R	TGGTCGTGGCTGATAAACCC	20
β-action-F	TGTAACCCAACAAGTGTCTT	20
β-action-R	CCACATACTGGCACTTTACT	20

**Table 2:** The identification of the duplicated type for *Sox* genes and all the genes in *C. japonica* and other representative species. WGD: whole-genome duplication.

Species		*C. japonica*	*G. gallus*	*C. virginianus*	*C. squamata*	*N. meleagris*	*B. thoracicus*	*M. gallopavo*
Singleton	Genome	3158	2443	4562	4491	4259	6043	5847
	*Sox*	0	0	0	0	0	0	0
Dispersed	Genome	4526	4353	12332	12266	4197	11588	6459
	*Sox*	13	12	13	14	9	13	9
Proximal	Genome	2015	4018	183	132	2580	91	1214
	*Sox*	0	1	1	0	1	0	0
Tandem	Genome	28974	37843	67	104	31830	41	19539
	*Sox*	4	3	0	0	5	0	4
WGD or segmental	Genome	416	560	22	139	375	0	263
	*Sox*	1	2	0	0	0	0	0
Total	Genome	39089	45199	17166	17132	43241	17763	33322
	*Sox*	18	18	14	14	15	13	13

**Table 3:** *Sox* family genes in *C. japonica* genome and their sequence characteristics. GRAVY: grand average of hydropathy.

Group	Gene	Intron Number	Length (aa)	Molecular Weight (KDa)	pI	GRAVY
B1	*Sox1*	0	373	37.91857	9.70	−0.505
	*Sox2*	0	404	44.21928	9.96	−0.641
	*Sox3*	0	316	34.02945	9.68	−0.715
B2	*Sox14*	0	240	26.66555	9.68	−0.630
	*Sox21*	0	280	28.76797	9.74	−0.218
C	*Sox4*	0	427	43.10559	7.10	−0.658
	*Sox11*	0	396	43.18391	4.92	−0.770
	*Sox12*	0	285	31.26546	7.65	−0.805
D	*Sox5*	21	773	85.07967	6.15	−0.746
	*Sox6*	15	816	90.57472	6.66	−0.817
	*Sox13*	15	612	68.31525	6.13	−0.810
E	*Sox8*	2	470	50.90391	6.37	−0.820
	*Sox9*	2	495	55.03546	6.16	−1.080
	*Sox10*	3	565	60.96868	7.47	−0.788
F	*Sox17*	1	387	40.38743	6.68	−0.580
	*Sox18*	1	418	46.22470	6.40	−0.791
	*Sox7*	1	377	41.15915	6.62	−0.759
H	*Sox30*	4	638	68.85069	6.07	−0.490

## Supplementary Materials

The following are available online at https://www.mdpi.com/2073-4425/10/4/314/s1, Figure S1: Heatmaps demonstrate *Sox* mRNAs between the incubation period and post-hatch period in the quail gonads. The color scale indicates the *Sox* mRNAs expression level, Table S1: Species of Galliformes genomes examined in this study as classified according to Clements et al. (2018), Table S2: Protein properties for *Sox* genes identified from *B. thoracicus* and *G. gallus*, Table S3: Ka/Ks analysis of between *Sox* gene pairs in quail, Table S4: Predicted numbers and locations of codons under positive selection within different *CjSox* genes, Table S5: GO enrichment analysis of *Sox* genes in quail.
